# Pharmacokinetic, Safety, and Immunogenicity Similarity of High‐ and Low‐Concentration Formulations of Adalimumab Biosimilar ABP 501, Adalimumab‐Atto

**DOI:** 10.1002/prp2.70236

**Published:** 2026-03-19

**Authors:** Vincent Chow, Muhan Zhou, Daniel T. Mytych, Alexander Colbert, Mieke Jill Miller, Iwona Wala, Ahad Sabet, Waldemar Radziszewski

**Affiliations:** ^1^ Amgen Inc. Thousand Oaks California USA; ^2^ Icon Salt Lake City Utah USA

**Keywords:** ABP 501, adalimumab, adalimumab‐atto, biosimilar, high‐concentration, pharmacokinetics, safety

## Abstract

ABP 501, a monoclonal anti‐tumor necrosis factor antibody, was the first adalimumab biosimilar approved in the US and EU as a 50 mg/mL formulation. An ABP 501 100 mg/mL high‐concentration formulation is also now approved. This study compares pharmacokinetics (PK), safety, and immunogenicity of the low‐ (ABP 501‐LCF) and high‐concentration (ABP 501‐HCF) formulations in healthy adults. This was a randomized, single‐blind, single‐dose, parallel‐group study in healthy adults. Participants were randomized to receive 40 mg of ABP 501 as HCF or LCF. Primary PK endpoints were area under the concentration‐time curve from time 0 extrapolated to infinity (AUC_inf_) and maximum observed concentration (C_max_) with a 90% confidence interval (CI) for the ratio of least square (LS) geometric means (GM) with a similarity margin of 0.80–1.25. Secondary endpoints included other PK parameters, safety, and immunogenicity. Baseline demographic and other characteristics were comparable between groups. Following study injection, ratios of LS GM (90% CIs) for AUC_inf_ and C_max_ between ABP 501‐HCF and ABP 501‐LCF were 1.04 (0.9634, 1.1297) and 1.06 (0.9960, 1.138), falling within the similarity margin. Both formulations were well tolerated with comparable adverse events. The occurrence of anti‐drug antibodies and neutralizing antibodies was comparable between groups. This study demonstrates PK similarity between ABP 501‐HCF and ABP 501‐LCF following a single subcutaneous injection in healthy adults. Immunogenicity and safety profiles were also similar. Given the totality of evidence of similarity of ABP 501 with adalimumab reference product (RP), it is anticipated that ABP 501‐HCF will perform similarly to the RP.

**Trial Registration:** NCT04270747.

## Graphical abstract

Consistent with previously demonstrated similarity of biosimilar ABP 501 (adalimumab‐atto) with adalimumab reference product, this study in healthy adults demonstrates the pharmacokinetic, safety, and immunogenicity similarity of a high‐concentration formulation (100 mg/mL) of ABP 501 with the original 50mg/mL formulation.

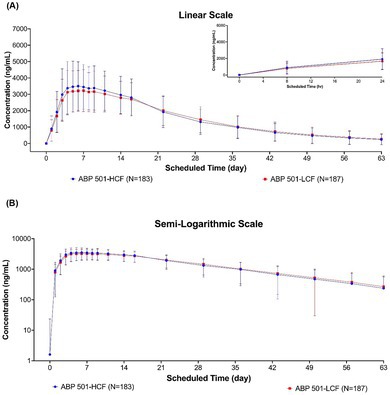

## 1 Introduction

ABP 501 (adalimumab‐atto) was developed as a biosimilar for Humira (adalimumab) and has been approved in the United States (US) (AMJEVITA), European Union (EU) (AMGEVITA), and in many regions around the world [1, 2]. Adalimumab is a recombinant, fully humanized monoclonal antibody that specifically binds to the inflammatory cytokine, tumor necrosis factor (TNF), a significant mediator of inflammation [3, 4]. By binding to TNF, adalimumab effectively neutralizes its biological activity, thereby reducing inflammation and its associated symptoms. Adalimumab is approved for the treatment of a range of inflammatory diseases [5, 6].

ABP 501 has the same amino acid sequence as adalimumab reference product (RP); data from analytical studies along with functional, pharmacokinetic (PK) assessment of similarity in healthy volunteers, and clinical comparisons of efficacy and safety in moderate‐to‐severe plaque psoriasis and moderate‐to‐severe rheumatoid arthritis indicate that ABP 501 is highly similar to adalimumab RP [7, 8, 9].

A new formulation of the adalimumab RP has been approved with a 50% reduced injection volume and corresponding higher concentration (40 mg/0.4 mL) than the prior formulation (40 mg/0.8 mL) [5, 6]. Accordingly, ABP 501 was developed and is now approved as a 100 mg/mL high‐concentration formulation in the US and EU [1, 2]. There are no changes to the drug substance or manufacturing process (same cell line) between the ABP 501 low‐concentration formulation (ABP 501‐LCF) and the ABP 501 high‐concentration formulation (ABP 501‐HCF). As with the RP, ABP 501‐HCF requires half the injection volume to deliver the same dose as ABP 501‐LCF. The notable excipient difference between the ABP 501‐LCF and ABP 501‐HCF formulations is the buffer system: acetate‐based in ABP 501‐LCF and lactate‐based in ABP 501‐HCF. This study was conducted to evaluate the comparability of single‐dose PK, safety, tolerability, and immunogenicity of ABP 501‐LCF and ABP 501‐HCF in healthy adult volunteers.

## 2 Methods

This was a randomized, single‐blind, single‐dose, 2‐arm, parallel‐group study in healthy adults. The study protocol and all accompanying materials were reviewed and approved by Advarra Institutional Review Board (PRO00054537). This study was conducted at 3 centres in the US in accordance with the Declaration of Helsinki and in compliance with the International Council for Harmonization (ICH) Good Clinical Practice (GCP) regulations/guidelines.

### 2.1 Study Population

Eligible participants were healthy adults between 18 and 55 years with normal or clinically acceptable physical examination, clinical laboratory test values, vital signs, and 12‐lead electrocardiogram. Participants were to have a body weight of ≥ 50 kg to ≤ 90 kg and a body mass index between 18.0 kg/m^2^ and 30.0 kg/m^2^. Key exclusion criteria were pregnancy, breastfeeding, or planning to become pregnant; a history of significant conditions, such as demyelinating disease or conditions affecting drug metabolism; recent bacterial, viral, parasitic, or fungal infections; a diagnosis of active or latent tuberculosis, surgery or major trauma within 3 months of screening; previously received adalimumab or a biosimilar of adalimumab.

### 2.2 Study Design

Screening procedures occurred within 35 days prior to dosing. Eligible participants were admitted to the Clinical Pharmacology Unit (CPU) and randomized in a 1:1 ratio to receive a single 40‐mg subcutaneous (SC) injection of either ABP 501 100 mg/mL (ABP 501‐HCF) or ABP 501 50 mg/mL (ABP 501‐LCF) in the upper left or upper right quadrant of the abdomen. Study drug was administered on Day 1 after predose baseline procedures were complete. Participants remained in the CPU and were discharged on Day 2 after the 24‐h postdose study procedures were completed. Participants returned to the CPU over the next 63 days for safety evaluations and blood sampling for PK and anti‐drug antibody (ADA) assessments (Figure S1; Table S1). The primary objective of the study was to determine the PK comparability of ABP 501‐HCF and ABP 501‐LCF following a single‐dose SC injection, as assessed principally by area under the serum concentration‐time curve from time 0 extrapolated to infinity (AUC_inf_) and maximum observed serum concentration (C_max_) in healthy adults. The secondary objective was to determine the comparative safety, tolerability, and immunogenicity.

### 2.3 Pharmacokinetics

Serum concentrations of ABP 501 were determined using a validated electrochemiluminescent assay [8]. The population for PK concentration analysis (PK Concentration Population) consisted of all participants who were randomized and who received any study drug and had at least 1 reported serum concentration value. Serum concentrations are summarized descriptively. Pharmacokinetic variables were calculated from the serum concentration data using noncompartmental methods (Phoenix WinNonlin) and actual sampling times. The PK Parameter Population consisted of all participants from the PK Concentration Population with an evaluable ABP 501‐HCF or ABP 501‐LCF serum concentration‐time profile. The PK parameters were estimated from the concentration‐time profiles, and areas under the curve (AUC) were calculated using the linear trapezoidal method. The primary endpoint of PK comparability of ABP 501‐HCF and ABP 501‐LCF was assessed using the ratio and 90% CIs of the least squares (LS) geometric mean (GM) of the serum PK parameters, AUC_inf_ and C_max_, calculated from an analysis of covariance (ANCOVA) model adjusted for baseline weight using the PK Parameter Population. PK comparability was established if the 90% CI of the LS GM ratios of AUC_inf_ and C_max_ were within the prespecified equivalence margin (0.8–1.25).

Subgroup and sensitivity analyses were conducted to examine a neutralizing ADA‐negative subgroup (PK parameter analysis set), as well as sensitivity analysis to explore the impact of gender using an ANCOVA model adjusted for gender in addition to baseline weight (PK parameter analysis set), and sensitivity analysis without adjusting for any covariates (PK parameter analysis set). Secondary PK endpoints examined were AUC from time 0 to the last quantifiable concentration (AUC_last_), time at which C_max_ is observed (T_max_), terminal elimination half‐life (t_½_), apparent clearance (CL/F), apparent volume of distribution (Vz/F), and mean residence time (MRT).

### 2.4 Safety and Immunogenicity

All safety analyses were performed on the Safety Population (randomized participants who received any amount of study drug) and summarized by time point and treatment using descriptive statistics. Safety assessments included physical examination, vital sign measurement, recording of concomitant medications and adverse events (AEs), clinical laboratory testing, and 12‐lead electrocardiogram (ECG). AEs were graded according to Common Terminology Criteria for Adverse Events (CTCAE) version 4.03. Events of interest (EOIs) prespecified for this study included serious infections, malignancies, hypersensitivity, demyelinating diseases, hematological reactions, heart failure, lupus‐like syndromes, liver enzyme elevations, and injection site reactions. The EOIs were retrieved utilizing Standardized Medical Dictionary for Regulatory Activities Queries (MedDRA, version 24.1) system organ class. The numbers and percentage of participants reporting any AE, serious AEs, and EOIs were tabulated by treatment and further classified by severity. Participants were only counted once; those with multiple events were counted under the category of their highest graded event. Blood samples were collected at Day 1 (predose), Day 16, Day 29, and at the end‐of‐study (EOS) visit (Day 63) and analyzed for ADAs. Samples positive for ADAs were further tested in neutralizing antibody (NAb) assessments. Results were summarized by treatment using the number and percentage of participants.

### 2.5 Determination of Sample Size

The sample size of 350 subjects (*n* = 175 per treatment group) was considered to provide a > 90% power at a two‐sided significance level of 0.05 to demonstrate comparability of primary PK endpoints based on the assumptions of between‐participant variability (as measured by coefficient of variation [CV]) of 53% for AUC_inf_ and C_max_ for ABP 501‐HCF and ABP 501‐LCF, true GM ratio of 1.05 between ABP 501‐HCF and ABP 501‐LCF, bioequivalence margin of (0.8–1.25), and a 5% drop‐out rate.

## 3 Results

### 3.1 Subject Disposition and Characteristics

**FIGURE 1:**
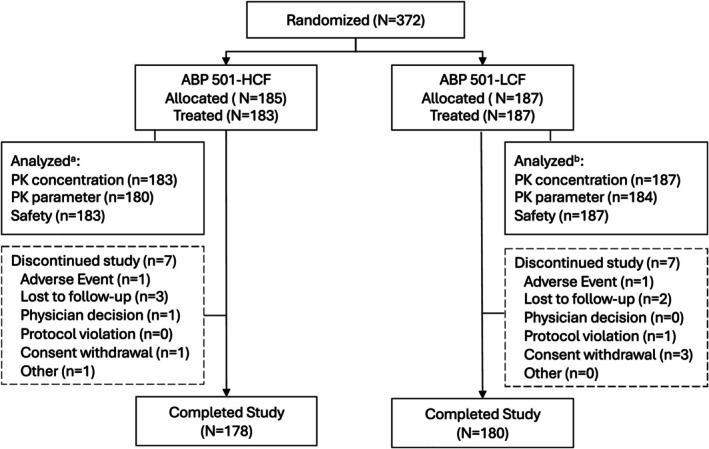
Study participant disposition. HCF, high‐concentration formulation; LCF, low‐concentration formulation; PK, pharmacokinetic. Baseline was defined as the last non‐missing assessment taken prior to the first dose of study investigational product. Percentages were calculated using the corresponding category count as the denominator. ^a^For the ABP 501‐HCF group, 2 subjects were excluded from the PK analysis set and the safety analysis set because they did not receive any study drug; 3 additional subjects were excluded from the PK parameter analysis set because they did not have an evaluable serum concentration‐time profile. ^b^For the ABP 501‐LCF group, 3 subjects were excluded from the PK parameter analysis set because they did not have an evaluable serum concentration‐time profile.

A total of 370 of 372 randomized participants received study drug (ABP 501‐HCF, 183 [98.9%]; ABP 501‐LCF, 187 [100.0%]). Overall, 358 (96.2%) participants completed the study (178 [96.2%] and 180 [96.3%], respectively) and 14 (3.8%) discontinued the study (7 [3.8%] and 7 [3.7%], respectively; Figure 1). Reasons for discontinuation included lost to follow‐up (5 [1.3%]), withdrawal (4 [1.1%]), and AEs (2 [0.5%]). Demographic and baseline characteristics were comparable between the 2 groups. Overall, 194 (52.4%) were male, 268 (72.4%) were white, and 188 (50.8%) were not Hispanic or Latino. The mean (SD) age was 34.8 (9.95) years with a range of 18 to 55 years.

### 3.2 Pharmacokinetics

**FIGURE 2:**
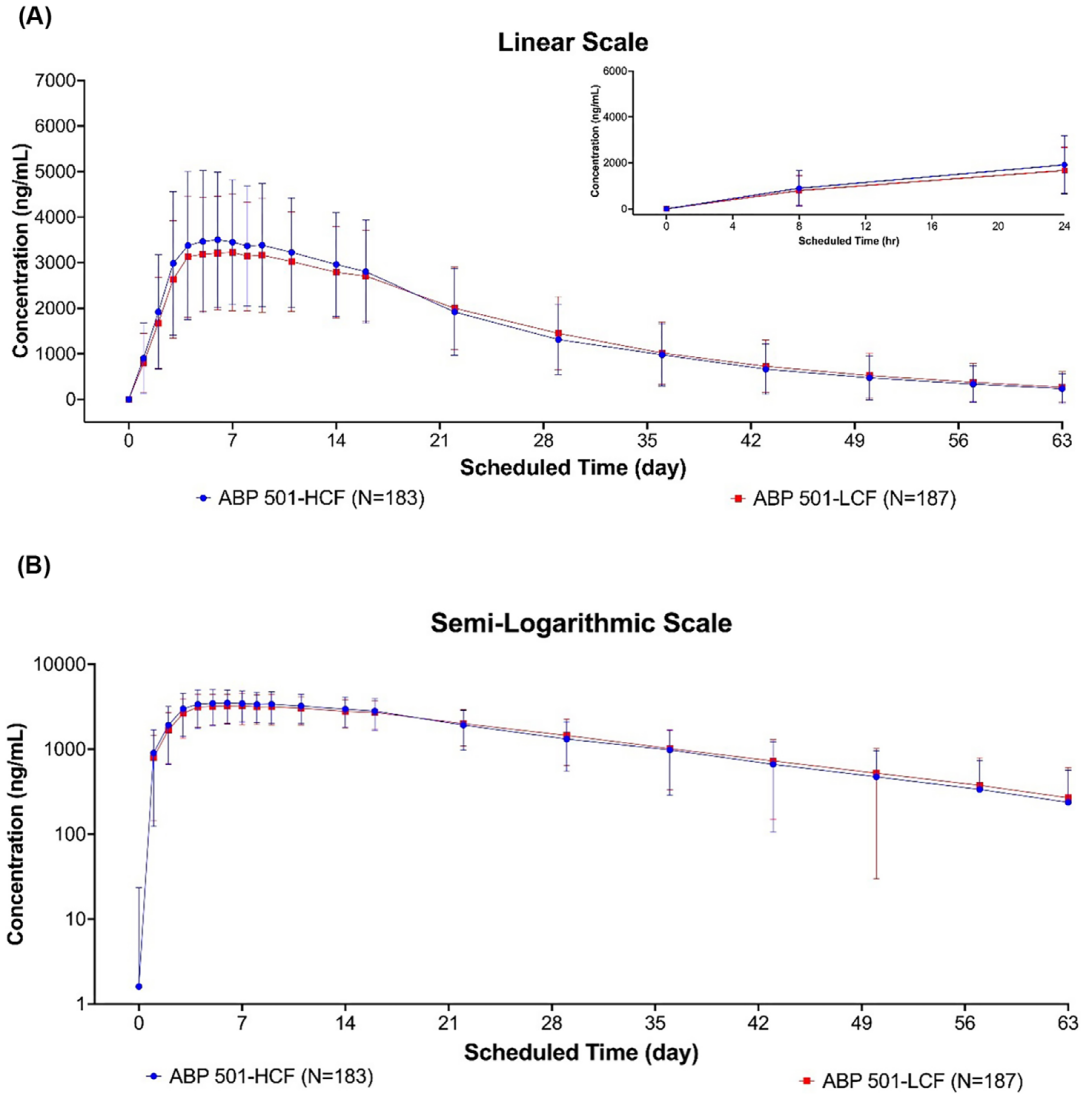
Mean (±SD) PK concentrations over time by treatment (PK concentration analysis set) on (A) linear and (B) semi‐logarithmic scale. HCF, high‐concentration formulation; LCF, low‐concentration formulation; PK, pharmacokinetic.

The mean serum concentration‐time profiles after a single SC injection of either study drug showed a high degree of similarity between the 2 groups (Figure 2). The descriptive statistical summary showed overall similar data between the three participating centres and between treatment groups overall. For both groups, peak concentrations were observed on approximately 6 days after injection, after which concentrations declined in a monophasic manner with a t_1/2_ of approximately 10 days. For the primary endpoint, resulting 90% CIs associated with the ratios of LS GM between ABP 501‐HCF and ABP 501‐LCF for AUC_inf_ and C_max_ were fully contained within the prespecified PK similarity margin (0.8–1.25; Table 1). ANCOVA with no adjustment for covariates had ratios of LS GM (90% CIs) between ABP 501‐HCF and ABP 501‐LCF for AUC_inf_ and C_max_ of 1.02 (0.9350, 1.1118) and 1.04 (0.9691, 1.1256). ANCOVA adjusted for baseline weight and gender as a sensitivity analysis measure, showed ratios of LS GM (90% CIs) between ABP 501‐HCF and ABP 501‐LCF for AUC_inf_ and C_max_ of 1.04 (0.9644, 1.1302) and 1.07 (0.9994, 1.1410). All 90% CIs were fully contained within the prespecified PK similarity margin (0.8–1.25). Pharmacokinetic secondary endpoints examined (AUC_last_, T_max_, t_1/2_, CL/F, Vz/F, and MRT) and their associated descriptive statistics were also similar between the treatment groups (Table 2).

**TABLE 1:** Summary of statistical assessment of PK parameters (PK parameter analysis set).

Treatment and comparison	AUC_inf_ (hr*μg/mL), LS geometric mean [*n*]	C_max_ (μg/mL), LS geometric mean [*n*]
ABP 501‐HCF	2177 [154]	3.75 [180]
ABP 501‐LCF	2086 [153]	3.52 [184]
**Ratio of LS geometric means (90% CI)**		
ABP 501‐HCF versus ABP 501‐LCF	1.04 (0.9634, 1.1297)	1.06 (0.9960, 1.1380)

*Note:* Primary analysis of geometric LS mean, ratio of geometric LS means, and 90% CI were estimated based on the ANCOVA model with a fixed effect for treatment and adjusting for baseline weight.

Abbreviations: ANCOVA, analysis of covariance; AUC, area under the concentration‐time curve; AUC_inf_, AUC from time 0 extrapolated to infinity; CI, confidence interval; C_max_, maximum observed concentration; HCF, high‐concentration formulation; LCF, low‐concentration formulation; LS, least squares; PK, pharmacokinetics.

**TABLE 2:** Summary of PK parameters (PK parameter analysis set).

	ABP 501‐HCF	ABP 501‐LCF
C_max_ (ng/mL) GM (Geo CV [%])	3710 (50.5)	3560 (40.0)
T_max_ (h) Median (range)	143.675 (24.00, 507.07)	144.775 (8.00, 626.62)
AUC_last_ (h*ng/mL) GM (Geo CV [%])	2 040 000 (49.2)	2 080 000 (45.1)
AUC_inf_ (h*ng/mL) GM (Geo CV [%])	2 150 000 (48.3)	2 110 000 (48.7)
t_1/2_ (h) Mean (SD)	245.558 (161.3392)	241.132 (146.8754)
AUC%Extrap (%) Mean (SD)	7.15 (8.956)	6.74 (7.845)
Vz/F (L) Mean (SD)	6.16 (3.37)	6.06 (2.89)
CL/F (L/h) Mean (SD)	0.0208 (0.0111)	0.0212 (0.0115)
MRT (h) Mean (SD)	482 (187)	495 (178)

*Note:* Geometric mean and Geo CV were only calculated for values > 0.

Abbreviations: AUC, area under the serum concentration‐time curve; AUC%Extrap, percentage of AUCinf due to extrapolation from the last quantifiable concentration observed to infinity; AUC_inf_, AUC from time 0 extrapolated to infinity; AUC_last_, AUC from time 0 to the last quantifiable concentration; CL/F, apparent clearance; C_max_, maximum observed serum concentration; Geo CV, geometric coefficient of variation; GM, geometric mean; HCF, high‐concentration formulation; LCF, low‐concentration formulation; MRT, mean residence time; PK, pharmacokinetic; SD, standard deviation; t_1/2_, terminal elimination half‐life; T_max_, time at which C_max_ is observed; Vz/F, apparent volume of distribution.

### 3.3 Safety and Immunology

The proportion of participants who experienced AEs was comparable between the 2 groups, 49 (26.8%) in the ABP 501‐HCF and 51 (27.3%) in the ABP 501‐LCF. All AEs were classified as CTCAE grade 1 or 2 in severity, with no grade ≥ 3, serious SEs, or fatal AEs reported. One participant in each group discontinued investigational product or discontinued the study due to an AE, which included COVID‐19 infection (Table 3). The most commonly reported AE in both treatment groups was headache (8 [4.4%] in the ABP 501‐HCF and 9 [4.8%] in the ABP 501‐LCF). There were no AEs with at least a 5% difference in frequency between treatment groups. The highest incidence rates of AEs were in the system organ class of infections and infestations (13 [7.1%] in ABP 501‐HCF and 8 [4.3%] in ABP 501‐LCF) and nervous system disorders (10 [5.5%] and 16 [8.6%], respectively). There were no system organ classes with at least a 5% difference between treatment groups. Prespecified EOIs were similar between the treatment groups (12 [6.6%] in ABP 501‐HCF and 11 [5.9%] in ABP 501‐LCF). The patient incidences for EOI hypersensitivity were 5 (2.7%) and 4 (2.1%), EOI hematological reactions were 1 (0.5%) and 0 (0.0%), for EOI liver enzyme elevations were 1 (0.5%) and 0 (0.0%), and for EOI injection site reactions were 5 (2.7%) and 8 (4.3%), respectively. There were no reported EOIs for serious infections, malignancies, demyelinating diseases, heart failure, or lupus‐like syndrome. There were no clinically impactful differences in laboratory values and vital signs between treatment groups or safety trends observed over time.

**TABLE 3:** Overall summary of adverse events (safety analysis set).

	ABP 501‐HCF (*N* = 183), *n* (%)	ABP 501‐LCF (*N* = 187), *n* (%)
Any AE	49 (26.8)	51 (27.3)
Any grade ≥ 3 AE	0 (0.0)	0 (0.0)
Any fatal AE	0 (0.0)	0 (0.0)
Any serious AE	0 (0.0)	0 (0.0)
Any AE leading to discontinuation of study	1 (0.5)	1 (0.5)
Any AE of interest	12 (6.6)	11 (5.9)
Any COVID‐19‐related AE	5 (2.7)	3 (1.6)

*Note:* Only treatment‐emergent adverse events were summarized by actual treatment received. For each category, participants were included only once, even if they experienced multiple adverse events in that category.

Abbreviations: AE, adverse events; HCF, high‐concentration formulation; LCF, low‐concentration formulation.

The overall incidence of participants with postdose ADAs to ABP 501 was comparable between the two groups (Table 4). All participants in the safety analysis set had at least 1 on‐study ADA result. Through EOS, 165 (90.7%) in ABP 501‐HCF and 171 (92.4%) in ABP 501‐LCF tested positive for the development of binding ADAs. Of the participants with a post‐baseline result through EOS, 31 (17.0%) in ABP 501‐HCF and 29 (15.7%) in ABP 501‐LCF tested positive for the development of neutralizing ADAs. A PK subgroup analysis examined participants who tested negative for neutralizing ADAs in the PK parameter analysis set. Statistical analysis comparing ABP 501‐HCF and ABP 501‐LCF for the subgroup showed that the ratios of LS GM and 90% CIs were within the equivalence margin (0.8 to 1.25) for AUC_inf_ (1.0383 [0.9599, 1.1230]), C_max_ (1.0728 [1.0052, 1.1450]), and AUC_last_ (0.9996 [0.9321, 1.0720]).

**TABLE 4:** Antidrug antibody results (safety analysis set).

Variable	ABP 501‐HCF (*N* = 183), *n* (%)	ABP 501‐LCF (*N* = 187), *n* (%)
**Subjects with an on‐study result** ^a^	183	187
*Total antibody incidence*, *n* (%)		
Binding antibody positive anytime	172 (94.0)	179 (95.7)
Neutralizing antibody positive anytime	32 (17.5)	29 (15.5)
**Subjects with a result at baseline**	183	187
*Pre‐existing antibody incidence*, *n* (%)		
Binding antibody positive at or before baseline	7 (3.8)	8 (4.3)
Neutralizing antibody positive at or before baseline	1 (0.5)	0 (0.0)
**Subjects with a post‐baseline result through EOS**	182	185
*Treatment boosted antibody incidence*, *n* (%)		
Binding antibody positive at baseline with a ≥ 4× increase in magnitude post‐baseline	4 (2.2)	6 (3.2)
*Developing antibody incidence*, *n* (%)		
Binding antibody positive post‐baseline with a negative or no result at baseline	165 (90.7)	171 (92.4)
Transient^b^	4 (2.2)	3 (1.6)
Neutralizing antibody positive post‐baseline with a negative or no result at baseline	31 (17.0)	29 (15.7)
Transient^b^	1 (0.5)	0 (0.0)

Abbreviations: EOS, end‐of‐study; HCF, high‐concentration formulation; LCF, low‐concentration formulation.

^a^ Participants were considered on‐study after signing informed consent.

^b^ Negative result at the participant's last time point tested within the study period.

## 4 Discussion

This randomized, single‐blinded, two‐arm, parallel‐group bridging study was designed to demonstrate PK comparability of ABP 501‐HCF and ABP 501‐LCF in healthy adults. Healthy volunteers were chosen as the study population to provide the most homogeneous sample for a sensitive comparison of the PK of ABP 501‐HCF with ABP 501‐LCF; this approach facilitates the interpretation of the study results with no confounding factors and/or concomitant medications. All participants received a single SC injection of 40 mg of ABP 501 of either the HCF (100 mg/mL) or LCF (50 mg/mL). The study results demonstrated that the 2 formulations exhibited similar PK characteristics as well as comparable safety and immunogenicity profiles between both treatments.

Pharmacokinetics (PK) comparability was established for ABP 501‐HCF and ABP 501‐LCF. The primary PK endpoints, AUC_inf_ and C_max_, were shown to be similar. The 90% CIs for the ratios of the LS GM were fully contained within the prespecified equivalence margin of 0.8 to 1.25. Results from subgroup and sensitivity analyses support the primary endpoint results of PK comparability between the 2 groups. Additionally, all secondary PK parameters were comparable between both treatment groups, consistent with the previous PK studies of ABP 501‐LCF which demonstrated PK equivalence of ABP 501 to both EU‐sourced and US‐sourced adalimumab RP [8].

Safety and tolerability were shown to be comparable between ABP 501‐HCF and ABP 501‐LCF groups. The proportion of participants that experienced treatment‐emergent AEs was similar in both groups. The most frequently reported common AEs related to study drug treatment by system organ class were infections and nervous system disorders, while headaches were the most common AE by preferred term. No serious AEs or significant safety concerns were observed. The incidence of ADAs was similar between groups, and the presence of anti‐ABP 501 NAbs did not lead to adverse reactions or safety events. While the presence of anti‐ABP 501 NAbs is not unexpected, their presence does not appear to be associated with any adverse treatment reaction or safety concern across both treatment groups. Additionally, examining PK within the neutralizing ADA negative subgroup demonstrated PK similarity between the ABP 501‐HCF and ABP 501‐LCF. Therefore, in general, safety profiles were found to be consistent with those already reported for ABP 501 and adalimumab, [5, 6, 8, 9] with no new safety concerns identified.

This study showed that ABP 501‐HCF and ABP 501‐LCF have similar PK, immunogenicity, and safety profiles after a single SC injection in healthy adults. The results support the conclusion of comparability between ABP 501‐HCF and ABP 501‐LCF. Given the demonstrated similarity between ABP 501 (adalimumab‐atto) and adalimumab RP based on the totality of evidence, including clinical studies, it is anticipated that there would be no clinically meaningful differences in efficacy or safety of ABP 501‐HFC compared to the reference product.

## Author Contributions

V.C., M.Z., D.T.M., A.C., M.J.M., I.W., and W.R. contributed to the study conception and design. A.S. collected the data. All authors contributed to the data analysis and interpretation of the data. All authors contributed to the drafting of the article and final approval of the submitted version.

## Funding

This work was supported by Amgen.

## Disclosure

Principal Investigator Statement: The authors confirm that the Principal Investigator for this study is Dr. Ahad Sabet and that he has direct clinical responsibility for study participants.

## Ethics Statement

Prior to initiation at each study canter, the study protocol, all amendments, the informed consent form and any accompanying materials provided were reviewed and approved by Advarra Institutional Review Board (PRO00054537). This study was conducted in accordance with the Note for Guidance on Good Clinical Practice (GCP) (International Council for Harmonization [ICH] Guideline E6 [R1] and 21 Code of Federal Regulations Parts 50, 56, and 312), the general principles indicated in the Declaration of Helsinki, and all applicable regulatory requirements.

## Consent

All subjects were to provide written informed consent prior to entering the study and before initiation of any study‐related procedure (including administration of investigational product). The investigator was responsible for explaining the benefits and risks of participation in the study to each subject or the subject's legally acceptable representative and for obtaining written informed consent.

## Conflicts of Interest

V.C., M.Z., D.T.M., A.C., M.J.M., I.W. and W.R. are employees and/or stockholders of Amgen Inc. A.S. declares no conflicts of interest.

## Supporting information


**Data S1:** Supporting Information.

## Data Availability

There is a plan to share data. This may include de‐identified individual patient data for variables necessary to address the specific research question in an approved data sharing request; also related data dictionaries, study protocol, statistical analysis plan, informed consent form, and/or clinical study report. Data sharing requests relating to data in this manuscript will be considered after the publication date and (1) this product and indication (or other new use) have been granted marketing authorization in both the US and Europe, or (2) clinical development discontinues and the data will not be submitted to regulatory authorities. There is no end date for eligibility to submit a data sharing request for these data. Qualified researchers may submit a request containing the research objectives, the Amgen product(s) and Amgen study/studies in scope, endpoints/outcomes of interest, statistical analysis plan, data requirements, publication plan, and qualifications of the researcher(s).In general, Amgen does not grant external requests for individual patient data for the purpose of reevaluating safety and efficacy issues already addressed in the product labelling. A committee of internal advisors review requests. If not approved, a Data Sharing Independent Review Panel may arbitrate and make the final decision. Requests that pose a potential conflict of interest or an actual or potential competitive risk may be declined at Amgen's sole discretion and without further arbitration. Upon approval, information necessary to address the research question will be provided under the terms of a data sharing agreement. This may include anonymized individual patient data and/or available supporting documents, containing fragments of analysis code were provided in analysis specifications. Further details are available at the following: http://www.amgen.com/datasharing.
