# Supplementary material for: Pharmacokinetic, Safety, and Immunogenicity Similarity of High‐ and Low‐Concentration Formulations of Adalimumab Biosimilar ABP 501, Adalimumab‐Atto
Source: Pharmacol Res Perspect. 2026 Mar 19;14(2):e70236. doi: 10.1002/prp2.70236 (PMC13140478; doi:10.1002/prp2.70236)
Supplement: Supplementary file 1 — Data S1: Supporting Information. [file PRP2-14-e70236-s001.pdf]

Pharmacokinetic, Safety, and Immunogenicity Similarity of High- and Low-Concentration Formulations of Adalimumab Biosimilar ABP 501, adalimumab-atto

Authors:

Vincent Chow[1], Muhan Zhou[1], Daniel T. Mytych[1], Alexander Colbert[1], Mieke Jill Miller[1], Iwona Wala[1], Ahad Sabet[2], and Waldemar Radziszewski[1]

Affiliations:

[1]Amgen Inc., Thousand Oaks, CA 91320; [2]ICON, Salt Lake City, UT 84124

Emails:

vchow@amgen.com, mzhou01@amgen.com, dmytych@amgen.com, acolbert@amgen.com, millermj@amgen.com, iwala@amgen.com, ahad.sabet@iconplc.com, wradzisz@amgen.com

Principal Investigator statement:

The authors confirm that the Principal Investigator for this study is Dr. Ahad Sabet and that he had direct clinical responsibility for study participants.

Keywords:

Biosimilar; Pharmacokinetics; Safety; ABP 501; High-concentration; Adalimumab

Supporting Information

FIGURE S1. Study Design.

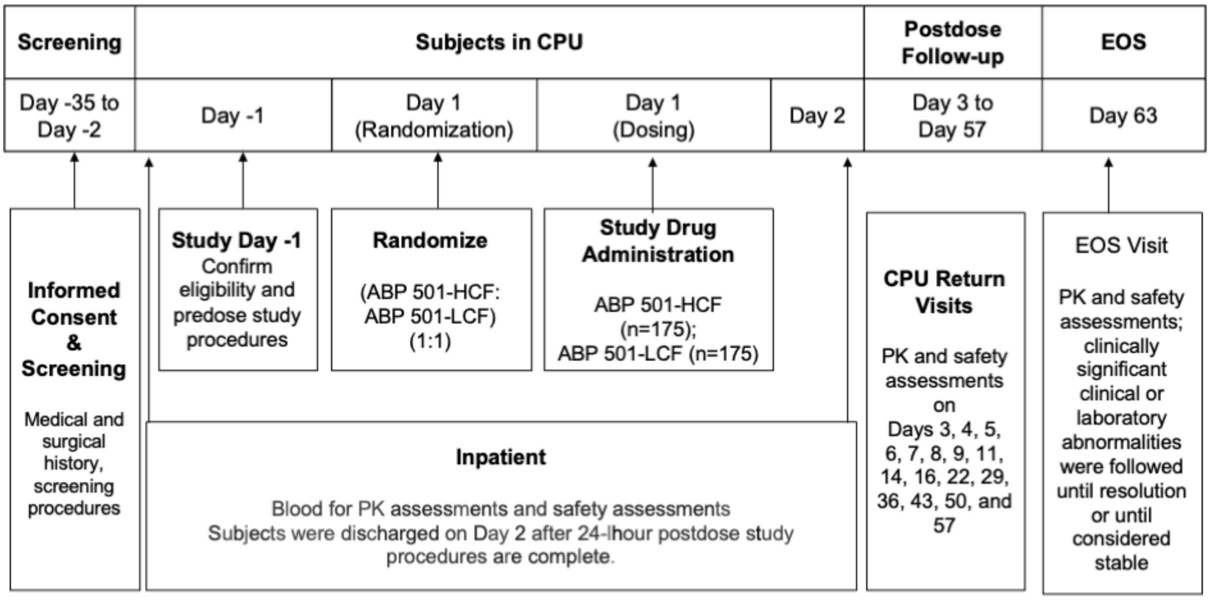

ABP 501-HCF = ABP 501 100 mg/mL; ABP 501-LCF = ABP 501 50 mg/mL; CPU = Clinical Pharmacology Unit; EOS = end-of-study; PK = pharmacokinetic; SC = subcutaneous

Notes: Planned dose: A single SC injection of ABP 501 100 mg/mL or ABP 501 50 mg/mL.
Subjects returned to the CPU for collection of blood for PK within the tolerance windows specified. Tolerance windows for return visits to the CPU and the EOS visit were consistent with tolerance windows for PK samples on these days.

TABLE S1. Schedule of Assessments.

	Screening	Treatment Period																			EOS
**Study Day[a]**	**-35 to -2**	**-1[b]**	**1[b]**	**2**	**3**	**4**	**5**	**6**	**7**	**8**	**9**	**11**	**14**	**16**	**22**	**29**	**36**	**43**	**50**	**57**	**63[c]**
Admission (A)/Confinement (C)/Discharge (D)/Return (R)		A	C	D	R	R	R	R	R	R	R	R	R	R	R	R	R	R	R	R	R
Informed consent	X																				
Review eligibility criteria	X	X	X																		
Medical and surgical history	X	X																			
Physical examination	X	X		X																	X
Vital signs	X	X	X[d]	X						X				X		X		X			X
Body weight	X	X																			X
Height	X																				
BMI	X	X[e]																			X
12-lead ECG	X[f]																				
Haematology	X	X		X						X				X			X				X
Chemistry	X	X		X						X				X			X				X
Urinalysis	X	X																			X
Pregnancy test[g]	X	X																			X
Serology (HBsAg, HBcAb, HCV, HIV)	X																				
TB test[h]	X																				
Chest X-ray[i]	X																				
Alcohol screen	X	X																			
Urine drug screen	X	X																			
Randomization			X																		
Study drug administration			X[j]																		
Antidrug antibody sampling			X[k]											X		X					X
PK sampling[l]			X[l]	X	X	X	X	X	X	X	X	X	X	X	X	X	X	X	X	X	X
AE monitoring			X	X	X	X	X	X	X	X	X	X	X	X	X	X	X	X	X	X	X
SAE reporting	X	X	X	X	X	X	X	X	X	X	X	X	X	X	X	X	X	X	X	X	X
Concomitant medications	X	X	X	X	X	X	X	X	X	X	X	X	X	X	X	X	X	X	X	X	X

AE = adverse event; BMI = body mass index; COVID-19 = coronavirus disease 2019; CPU = Clinical Pharmacology Unit; CT = computed tomography; ECG = electrocardiogram; EOS = end-of-study; HBcAb = hepatitis B core antibody; HBsAg = hepatitis B surface antigen; hCG = human chorionic gonadotropin; HCV = hepatitis C virus; HIV = human immunodeficiency virus; IGRA = interferon gamma releasing assay; MRI = magnetic resonance imaging; PK = pharmacokinetic; SAE = serious adverse event; TB = tuberculosis.

Note: Additional COVID-19 related precautions and procedures (including SARS-CoV-2 testing/screening) were implemented based on the prevailing situation during study conduct, at the investigator's discretion.

[a] Subjects returned to the CPU for collection of blood for PK as per study protocol.

[b] Assessments conducted on Day 1 and predose on Day 1 were used to re-confirm a subject's eligibility for enrollment into the study.

[c] EOS evaluations were performed prior to study discharge or for subjects who were prematurely discontinued.

[d] Vital signs (temperature, respiratory rate, supine blood pressure, and pulse) were measured predose and one-hour postdose on Day 1. Blood pressure and heart rate (pulse) were measured after the subject had been resting quietly in a supine position or in the most recumbent position possible for at least 5 minutes.

[e] BMI was calculated using the height obtained at screening; the BMI on Day 1 was used to re-confirm a subject's eligibility for enrollment into the study.

[f] Electrocardiograms were recorded after the subject had been resting quietly in a supine position or in the most recumbent position possible for at least 5 minutes. Triplicate screening ECG was performed to obtain mean with the full set of 3 ECG tracings completed within 6 minutes.
[g] A serum hCG test at screening and serum or urine hCG test on Day 1 and EOS.
[h] A QuantiFERON® TB Gold test or local IGRA equivalent (eg, T-Spot® test was performed. TB testing may have been repeated during the course of the study at the discretion of the investigator.
[i] A chest X-ray (posterior-anterior and lateral views) was performed at screening in subjects who had not previously had a chest X-ray (or chest CT-scan or MRI) within 3 months of screening. Historical films obtained or formal reports signed off by radiologist in the 3 months prior to screening were acceptable. Women must have had a negative pregnancy test (performed at screening) before the chest X-ray was performed.
[j] ABP 501 100 mg/mL or ABP 501 50 mg/mL was administered subcutaneously in the upper left or upper right quadrant of the abdomen.
[k] The antidrug antibody sample was collected predose on Day 1.
[l] PK sample for Day 1 includes sample collection at predose and 8 hours postdose.
